# Incidence and Risk Factors for Developing Type 2 Diabetes Mellitus After Acute Myocardial Infarction—A Long-Term Follow-Up

**DOI:** 10.3390/jcdd12030089

**Published:** 2025-02-28

**Authors:** Tamara Yakubov, Muhammad Abu Tailakh, Arthur Shiyovich, Harel Gilutz, Ygal Plakht

**Affiliations:** 1 Department of Nursing, Recanati School for Community Health Professions, Faculty of Health Sciences, Ben-Gurion University of the Negev, Beer Sheva 84101, Israel; yakubovtamara@gmail.com (T.Y.); abutaila@bgu.ac.il (M.A.T.); 2 Department of Internal Medicine E, Soroka University Medical Center, Beer Sheva 84101, Israel; 3 Nursing Research Unit, Soroka University Medical Center, Beer Sheva 84101, Israel; 4 Department of Cardiology, Rabin Medical Center, Petach Tikva 49414, Israel; arthur.shiyovich@gmail.com; 5 Faculty of Medical and Health Sciences, Tel Aviv University, Tel Aviv 69978, Israel; 6 Division of Cardiovascular Medicine, Department of Medicine, Brigham and Women’s Hospital, Harvard Medical School, Boston, MA 02115, USA; 7 Goldman Medical School, Faculty of Health Sciences, Ben-Gurion University of the Negev, Beer Sheva 84101, Israel; gilutz@bgu.ac.il; 8 Department of Emergency Medicine, Soroka University Medical Center, Beer Sheva 84101, Israel

**Keywords:** acute myocardial infarction, type 2 diabetes mellitus, new-onset diabetes mellitus, incidence, long-term follow-up

## Abstract

Acute myocardial infarction (AMI) and type 2 diabetes mellitus (T2DM) share common risk factors. To evaluate the long-term incidence and predictors of new-onset T2DM (NODM) among post-AMI adults, we conducted a retrospective analysis of AMI survivors hospitalized between 2002 and 2017. Eligible patients were followed for up to 16 years to identify NODM, stratified by demographic and clinical characteristics. Among 5147 individuals (74.2% males, mean age 64.6 ± 14.9 years) without pre-existing T2DM, 23.4% developed NODM (cumulative incidence: 0.541). Key risk factors included an age of 50–60 years, a minority ethnicity (Arabs), smoking, metabolic syndrome (MetS), hemoglobin A1C (HbA1C) ≥ 5.7%, and cardiovascular comorbidities. A total score (TS), integrating these factors, revealed a linear association with the NODM risk: each 1-point increase corresponded to a 1.2-fold rise (95% CI 1.191–1.276, *p* < 0.001). HbA1C ≥ 6% on the “Pre-DM sub-scale” conferred a 2.8-fold risk (*p* < 0.001), while other risk factors also independently predicted NODM. In conclusion, post-AMI patients with multiple cardiovascular risk factors, particularly middle-aged individuals, Arab individuals, and those with HbA1C ≥ 6% or MetS, are at a heightened risk of NODM. Early identification and targeted interventions may mitigate this risk.

## 1. Introduction

Type 2 diabetes mellitus (T2DM) is a chronic condition, due to a non-autoimmune progressive loss of adequate β-cell insulin secretion, frequently against a background of insulin resistance and metabolic syndrome (MetS) [1]. T2DM is a well-established risk factor for coronary heart disease and particularly acute coronary syndrome (which includes ST-elevation myocardial infarction [STEMI] and non-ST-elevation acute coronary syndrome [NSTE-ACS], with the sub-categories of non-ST-elevation myocardial infarction [NSTEMI] and unstable angina) [2] and is associated with poor prognoses [3,4,5,6,7,8]. However, limited data exist regarding the incidence and risk factors for new-onset T2DM (NODM) in patients who have experienced acute myocardial infarction (AMI) [9,10,11]. Previous studies have identified several potential contributors to post-AMI NODM, such as MetS and pre-DM [12,13,14,15,16,17]. Despite these observations, the specific mechanisms and pathways linking AMI to diabetes onset remain incompletely understood. Notably, post-AMI recovery involves immune and neurohormonal activation, which can promote insulin resistance and NODM development [18,19,20]. In addition, medication adherence [21,22,23,24,25,26,27,28] and lifestyle factors, including physical inactivity and suboptimal dietary habits, significantly affect long-term outcomes and increase the risk of NODM in AMI patients [29,30,31,32,33,34,35,36,37,38].

Given these insights, there remains a pressing need for further research into NODM following AMI, particularly with respect to associated risk factors across diverse patient populations. This study aims to evaluate the incidence of NODM in adults post-AMI and to identify the risk factors contributing to its development.

## 2. Materials and Methods

### 2.1. Study Population and Outcomes

This retrospective study was conducted as part of the Soroka Acute Myocardial Infarction (SAMI) project at the Soroka University Medical Center (SUMC) [39,40]. The SUMC is a tertiary teaching hospital with approximately 1200 beds, serving the southern district of Israel, and is the second largest hospital in the country. Over 500,000 residents live in southern Israel; approximately 35% of them are Muslim Arabs (Bedouins). Despite the geographic proximity, these ethnic groups greatly differ in their lifestyle, demographic growth, morbidity, and health-related outcomes [41,42,43].

The study cohort comprised adult patients (aged 18 years and older) who were discharged alive from the SUMC with a diagnosis of AMI from 2002 to 2017. The exclusion criteria were a pre-existing recorded diagnosis of DM at the time of study enrollment or confirmed by laboratory assessments (two hemoglobin A1C [HbA1C] test results of ≥6.5% or two random blood glucose levels of ≥200 mg/dL), NODM within one year post-discharge, foreign workers, an absence of essential data, and mortality during hospitalization or within one year post-discharge. To prevent duplicate cases, for patients with multiple hospitalizations at the SUMC, only the first hospitalization was considered for the analysis. This study received institutional ethical approval (approval number SOR-0319-16), waiving the need for patient consent due to its retrospective nature. All data handling complied with strict confidentiality and information security protocols, ensuring that no personal identifiers were accessible to the researchers.

The follow-up duration encompassed the period beginning one year after discharge from the index hospitalization and extending for up to 16 years (or 31 July 2023, whichever occurred first). NODM was designated as the primary outcome and was determined using the following criteria: the registration of a type 2 DM (T2DM) diagnosis (by the International Classification of Diseases, Ninth Revision, Clinical Modification [ICD-9-CM] codes 250*) or laboratory results meeting the 2023 criteria of the American Diabetes Association (ADA) [1]. According to this definition, any patients with two HbA1C test results of ≥6.5% or two random blood glucose test results of ≥200 mg/dL were classified as having NODM. To evaluate the risk of developing NODM, the patients were categorized based on HbA1C levels as follows: no available HbA1C results, <5.7% (normal), 5.7–6.0% (pre-DM, lower risk) and ≥6.0% (pre-DM, higher risk). These categories were used to assess which HbA1C ranges were associated with an increased risk of NODM. Patients who died during the follow-up period (with no NODM) were defined as censored. The study design chart is presented in the Supplementary Materials Figure S1.

### 2.2. Data Collection and Definitions

Baseline data were obtained from the computerized medical records of the SUMC database. As previously reported, the collected data included demographics, comorbidities, laboratory results, echocardiographic and angiographic findings, and AMI management [44]. The follow-up data were retrieved from electronic medical records of SUMC and primary clinics. We utilized the ICD-9-CM codes to identify comorbidities, as recorded by the attending medical staff during patient hospitalization (Supplementary Materials Table S1).The index hospitalization refers to the primary hospital admission diagnosed with AMI based on ischemic signs and/or symptoms coupled with an abrupt rise and fall in cardiac biomarkers levels consistent with acute myocardial injury [45], (ICD-9-CM codes 410*). In addition to ICD-9-CM criteria, dyslipidemia was defined as low-density lipoprotein (LDL) levels ≥ 100 mg/dL at any time point throughout the 12 months preceding and following hospitalization [46]. Obesity was defined as a body mass index (BMI) of ≥30 kg/m^2^ [47], and the definition of anemia was based on a blood Hb level of <13 mg/dL in men or <12 mg/dL in women [48]. Significant coronary artery disease was defined as the detection of ≥70% vessel stenosis on angiography. Severe left ventricular dysfunction was defined as an ejection fraction of <30% on the first echocardiogram conducted during hospitalization; a pulmonary arterial systolic pressure of ≥37 mmHg on the same exam indicated pulmonary hypertension. Valvular heart diseases (mitral and tricuspid regurgitation) were referred to as being of moderate and above severity, as graded by experienced echocardiologists and based on the American Society of Echocardiography guidelines [49].

### 2.3. Statistical Analysis

Statistical analyses were performed using IBM SPSS version 29 software. Quantitative variables were summarized using means and standard deviations (SDs) and medians and interquartile ranges (IQRs), while nominal variables were presented as counts (*n*) and percentages. The incidence of NODM during follow-up was determined using the Kaplan Meier survival analysis. A multivariate Cox regression analysis was conducted to identify the risk factors for NODM, incorporating relevant variables that exhibited a statistically significant relationship with the outcome. The results of this analysis were presented as adjusted hazard ratios (AdjHRs) with 95% confidence intervals (CIs). Additionally, we developed a risk score to quantify the risk of developing NODM based on the AdjHR values, with scores exceeding 1.2 being converted into a total weighted score (TS), as exemplified in the Charlson Comorbidity Index [50]. Subsequently, a TS was calculated for each subject, considering multiple parameters, and the hazard ratio (HR) was employed to analyze the relationship between these total scores and the outcome. Two sub-scales were created: the “Pre-DM sub-scale” and the “Other risk factors sub-scale”, which included other risk factors, such as demographics and cardiovascular and metabolic conditions. A significance threshold of *p* < 0.05 (two-tailed) was applied to all the tests.

## 3. Results

### 3.1. Baseline Characteristics of the Study Population

From the initial patient cohort available for research, 5147 patients were qualified for our study (Supplementary Materials Figure S2). Table 1 summarizes the population’s baseline characteristics. The mean age of the subjects was 64.60 years (SD = 14.90). Most of the participants were male and Jewish. Smoking and hypertension were the most prevalent among the cardiovascular risk factors. About 11% of the study cohort had a documented history of MI, and more than 10% had neurological disorders—42.4% of them (*n* = 236) had a recorded history of stroke. About 10% of the patients were classified as being pre-DM (HbA1C ≥ 5.7%). Regarding the type of AMI, more than half of the cases were STEMI. The most common intervention employed was cardiac catheterization.

### 3.2. Follow-Up and Outcome

The follow-up duration ranged from one year to approximately 15.8 years, with a median of 5.08 years (IQR 2.79–8.58 years). Over this period, 1202 patients (23.4%) developed NODM, while 2050 individuals (39.8%) died. The cumulative incidence of NODM during the follow-up period was 0.541.

### 3.3. The Cumulative Incidence of NODM by the Baseline Characteristics

Table 2 presents the cumulative incidence of NODM during the follow-up period across various examined parameters. Significant differences in the NODM incidence were observed among the different age groups, suggesting that the younger patients (<50 years group) in the post-AMI population were at a higher risk. A higher NODM incidence was observed in the Arab individuals compared to the Jewish individuals. A higher cumulative NODM incidence was observed in the patients with peripheral vascular disease (PVD), hypertension, obesity, or dyslipidemia. The participants without recorded laboratory and BMI results exhibited the highest NODM incidence. Additionally, a pre-DM status and the absence of HbA1C recorded results exhibited an elevated cumulative NODM incidence.

### 3.4. The Risk of Developing NODM Based on the Investigated Parameters—Multivariable Analysis

The results of the multivariable analysis (Table 3) demonstrated that the patients aged 50–60 years and Arab individuals had an increased risk of developing NODM compared to individuals under 50 years and Jewish individuals, respectively.

Significant risk factors for developing NODM included the presence of cardiomegaly, a history of MI, an atrioventricular (AV) block, hypertension, smoking, PVD, and obesity. Additionally, NSTEMI vs. STEMI and mitral valve regurgitation were associated with an elevated risk of developing NODM. Dyslipidemia and HbA1C levels of above 5.7% were also associated with a higher risk of NODM. Moreover, the patients with HbA1C levels ≥ 6% exhibited the highest risk for NODM, with an AdjHR of 3.346 (95% CI: 2.353–4.760; *p* < 0.001).

### 3.5. Risk Scoring

After computing the weighted values for each parameter based on the results of the multivariable model (Supplementary Materials Table S2), the TS ranged from 0 to 11, with a mean of 3.33 (SD = 1.75). Half of the subjects scored ≥3 points. Due to the relatively low number of patients in some groups, scores of 0 and 1 were combined, as were scores of ≥7. Given the strong association between being pre-DM and the risk of NODM, two sub-scales were created: the “Pre-DM sub-scale” (for HbA1C levels ≥ 5.7%) and the “Other risk factors sub-scale”, which consisted of the age, ethnicity, LDL level, hypertension, BMI, smoking, cardiomegaly, history of MI, type of AMI, atrioventricular block, peripheral vascular disease, and mitral regurgitation. Supplementary Materials Figure S3 presents the scores for these sub-scales. Importantly, a high percentage of patients with a pre-DM status had high levels of the TS. For instance, among the participants with ≥7 points, more than half (53.2%) had a pre-DM status.

Patients with a higher TS showed a progressively increased risk of NODM, with those scoring ≥7 having a significantly higher risk compared to those with scores of ≤1 (HR = 4.136; 95% CI: 3.168–5.400; *p* < 0.001). A direct linear relationship was observed, where each additional point in the TS increased the risk of NODM by approximately 23% (HR = 1.225; 95% CI: 1.188–1.263; *p* < 0.001).

**Figure 1:**
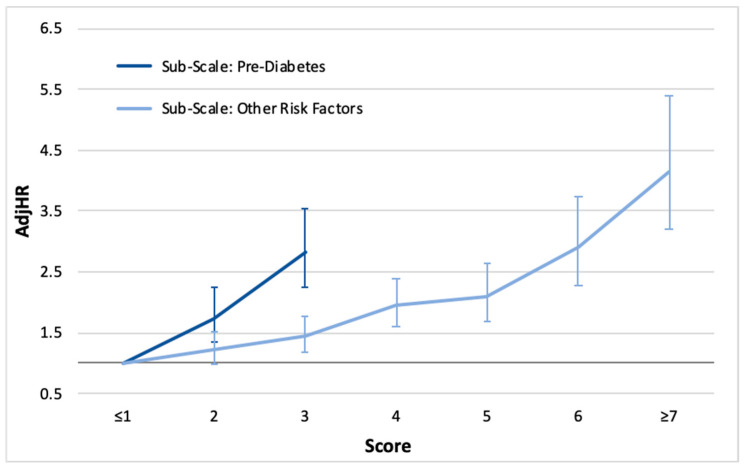
The relative risk (hazard ratios with 95% confidence intervals) for new-onset type 2 diabetes mellitus, by the scores of the “Pre-diabetes Sub-Scale” and the “Other Risk Factors Sub-Scale”. The “Pre-diabetes sub-scale” refers to hemoglobin A1C levels of ≥5.7%. The “Other risk factors sub-scale” consisted of the age, ethnicity, low-density lipoprotein, hypertension, body mass index, smoking, cardiomegaly, history of myocardial infarction, type of acute myocardial infarction, atrioventricular block, peripheral vascular disease, and mitral regurgitation (see Supplementary Materials Table S2). AdjHR—adjusted hazard ratio.

The relative risk for NODM, in accordance with the values of the “Pre-DM sub-scale” and the “Other risk factors sub-scale”, is presented in Figure 1. Patients scoring 2 or 3 points on the “Pre-DM sub-scale” (HbA1C of 5.7–6.0% and ≥6.0%, respectively) had an NODM risk of 1.739 (95% CI: 1.340–2.255) and 2.812 (95% CI: 2.235–3.538), respectively (*p* < 0.001 for each). Additionally, each point increase in the “Other risk factors sub-scale” was associated with a 1.2-fold higher risk of NODM (AdjHR = 1.191; 95%: CI 1.151–1.232; *p* < 0.001).

## 4. Discussion

The study aimed to assess the incidence of NODM following AMI and to identify the associated risk factors over a follow-up period of up to 16 years. The main findings of the study were as follows: (1) the cumulative incidence of NODM in the study population was 54%; (2) the risk factors for developing NODM included demographic factors (individuals aged 50–60 years; Arab individuals), cardiovascular risk factors (smoking, MetS, and pre-DM), cardiovascular diseases, and patients who experienced NSTEMI; and (3) the risk of developing NODM increased with the number of risk factors present.

### 4.1. HbA1C Is the Strongest Predictor

The majority of the patients were pre-DM on admission or had borderline high HbA1C. Thus, it is unsurprising that our study’s cumulative incidence of NODM was 54%. Indeed, a high HbA1C level is the strongest predictor of post-AMI NODM. Globally, the progression from pre-DM to T2DM is around 5–10% each year, and eventually, diabetes develops in up to 70% of patients during a lifetime [51,52]. However, in subjects without DM, HbA1C values differ slightly in different ethnic groups, which might impact the HbA1C point in diagnosing DM [53].

### 4.2. “No Results” Phenomenon

Notably, the absence of HbA1C results, although not statistically significant in predicting the NODM risk, still reflects a critical issue—poor adherence to basic secondary prevention measures, including MetS screening and post-AMI care. In our study, 4.2% of patients with missing HbA1C data were diagnosed with T2DM based on blood tests, highlighting the risk of missed diagnoses. This was similar to findings in large-scale studies in the US, which found that the pre-DM screening rates were low (62.8%), the documentation was inadequate (5.4%), and there was no appropriate treatment of pre-DM [54]. Similarly, poor glycemic control, indicating sub-optimal care for diabetes and other comorbidities, was documented in Iran [55]. It should be mentioned that low adherence is dependent both on the patient and his caregiver [56]. In this line, our study demonstrated that being Arab was found to be a risk factor in developing NODM. Poor adherence with treatment in that ethnic group was 66% for hypertension and 72.9% for T2DM [57]. Health disparities, including difficulties in accessing healthcare services for preventive purposes, further contribute to this ethnicity-dependent low adherence [43,58].

### 4.3. The Cause and Effect of Risk Factors on NODM

The predictors of NODM that were statistically calculated may be a result or a cause of hyperglycemia, potentially leading to NODM. Some of these predictors have a common final pathway causing insulin resistance. It seems that obesity, dyslipidemia, hypertension, and smoking are well-established components of MetS, all of which may induce chronic inflammation and immune system activation, producing reactive oxygen species (ROS) and other inflammatory mediators, prompting insulin resistance [59,60,61,62,63,64,65,66,67,68]. Additionally, PVD shares common risk factors and may contribute to the development of insulin resistance and the progression toward NODM [69].

Mitral regurgitation, cardiomegaly and AV blocks were identified as cardiovascular predictors of NODM, which may represent subclinical heart failure or diabetic cardiomyopathy (DCM) [70]. An association between insulin resistance and heart failure was demonstrated in an animal model [71]. Conversely, it was suggested that inflammation and insulin resistance may produce adverse cardiovascular outcomes, inducing cardiovascular structural and functional disturbances [72]. NSTEMI predicted NODM, probably representing older patients with multimorbidity [73,74]. The sequela of these network pathways may lead to NODM.

The excess mortality of diabetic patients results from congestive heart failure caused by DCM, severe coronary artery disease, the decreased vasodilatory reserve of epicardial and resistance arteries, and possibly the abnormal metabolism of myocardial substrate [75]. DCM is a specific form of cardiac dysfunction that occurs in the setting of diabetes, independent of other known cardiac diseases [76,77]. The risk of developing DCM is significantly higher in individuals with long-standing DM and poor glycemic control [76]. However, impaired left and right atria dysfunctions were demonstrated also in pre-diabetic patients [77]. A common pathway leading to DCM and atherosclerosis increases in reactive oxygen species production in diabetic cardiovascular cells was suggested [78]. Importantly, an increased risk for cardiac events was associated with myocardial fibrosis, as demonstrated using late gadolinium enhancement in cardiac magnetic resonance imaging (MRI), potentially presenting silent myocardial infarction [79]. We believe that the early initiation of antidiabetic medical therapy together with novel pioneering therapeutical strategies (like gene therapy and non-coding RNA) [76] could minimize the risk of DCM for post-AMI NODM patients.

### 4.4. Total Score

We calculated a TS for each patient, according to the number of risk factors and their strength of association with the outcome. The average TS was 3.33 ± 1.75; half of the patients scored ≥3 points. The patients with higher scores showed a progressively increased risk of NODM with a direct linear relationship. For each additional point in the risk score, the diabetes risk increased by 23.3%.

That represents a high-risk population, probably due to high levels of HbA1C in 67% of the patient cohort. During the follow-up period, 23.4% developed NODM, yet 39.8% died, some of which might have developed NODM. That might partially explain the under-expected low rate of progression.

The TS is a valuable tool to identify the population at a very high risk of developing T2DM, requiring immediate and intense post-AMI and long-term intervention. The “Pre-DM sub-scale” reinforced the strong link between being pre-DM (HbA1C of 5.7–6.0% and ≥6.0%) and a higher NODM risk, highlighting the need for effective monitoring and management of HbA1C levels.

### 4.5. Limitations

There are several limitations in our study. It was a single-center study conducted under a retrospective design, which limits the ability to capture all diagnostic and treatment variables. However, it is a “real-life” database, unbiased by research studies that tend to exclude certain patients.

In our study, some significant factors were unavailable: medical treatment, adherence with guidelines, a sedentary lifestyle, and diet. “No results” is a surrogate marker of non-adherence; the same is true for a high BMI or not performing an echocardiogram after AMI. The magnitude of the missing data may be a strong bias, which is challenging to overcome when using a real-life database. The ethnic differences in HbA1C “normal” levels are some potential biases.

## 5. Conclusions

To the best of our knowledge, this is the first study to examine the risk factors of NODM following AMI over a long-term follow-up period based on a large, “real-life” population. Our findings emphasize the heightened risk of long-term NODM development among post-AMI patients, particularly among middle-aged individuals, minorities, and those with rich comorbidities, notably MetS. This highlights the importance of integrating comprehensive DM screening and preventative strategies into post-AMI care protocols, particularly for those with the significant risk factors identified in this study. The early detection and management of NODM post-AMI are crucial due to associated risks of mortality, recurrent MI, stroke, and recurrent catheterization within the first year.

Future research directions may include a prospective follow-up study focusing on investigating various outcomes (e.g., cardiovascular and non-cardiovascular mortality, major adverse cardiovascular and cerebrovascular events [MACCE], etc.) among the patients who developed NODM compared to those who did not develop NODM after AMI. Additionally, further population-based research could incorporate the general population (i.e., individuals without AMI) to determine the relative contribution of an AMI event to the development of diabetes. Such a study could also explore additional parameters, including a range of biomarkers, pharmacological treatments, and lifestyle changes, over the follow-up period.

## Figures and Tables

**Table 1:** Baseline characteristics of the study patients (*n* = 5147).

Parameter	Category	*n* (%)
**Demographics**		
Age, years	<50	1017 (19.8)
	50–60	1220 (23.7)
	≥60	2910 (56.5)
Sex	Male	3818 (74.2)
Ethnicity	Minorities (Arabs)	829 (16.1)
**Cardiac diseases**		
Cardiomegaly		367 (7.1)
Atrial fibrillation/flutter		749 (14.6)
CHF		546 (10.6)
Chronic pulmonary heart disease		307 (6.0)
History of MI		580 (11.3)
AV block		165 (3.2)
**Cardiovascular risk factors**		
Renal diseases		244 (4.7)
Smoking		2434 (47.3)
PVD		415 (8.1)
Hypertension		2384 (46.3)
BMI, kg/m^2^	No results	3704 (72.0)
	<30	1068 (74.1)
	≥30	375 (25.9)
**Results of laboratory tests**		
HbA1C baseline, %	No results	4119 (80.0)
	<5.7	528 (10.3)
	5.7–6.0	279 (5.4)
	≥6.0	221 (4.3)
LDL, mg/dL	No results	560 (10.9)
	<100	2031 (44.2)
	≥100	2556 (55.7)
Total cholesterol, mg/dL	No results	361 (7.0)
	<200	3523 (73.6)
	200–240	903 (18.8)
	≥240	360 (7.5)
HDL, mg/dL	No results	886 (17.2)
	<40	2054 (48.2)
	40–60	1899 (44.5)
	≥60	308 (7.2)
Triglycerides, mg/dL	No results	370 (7.2)
	<150	3008 (63.0)
	150–200	991 (20.7)
	200–500	745 (15.5)
	≥500	33 (0.7)
**Other disorders**		
COPD		334 (6.5)
Neurological disorders		557 (10.8)
Malignancy		139 (2.7)
Anemia		1881 (36.5)
**Clinical characteristics of AMI**		
Type of AMI	STEMI	2788 (54.2)
**Results of echocardiography** (***n* = 4415, 85.8%**)		
Severe LV dysfunction		343 (7.8)
LV hypertrophy		170 (3.9)
Mitral regurgitation		190 (4.3)
Tricuspid regurgitation		124 (2.8)
Pulmonary hypertension		222 (5.0)
**Results of angiography** (***n* = 3971, 77.2%**)		
Measure of CAD	No or non-significant	176 (4.4)
	One vessel	1222 (30.8)
	Two vessels	1157 (29.1)
	Three vessels/LM	1416 (35.7)
	No results	1176 (29.6)
**Type of treatment**		
Type of treatment	Noninvasive	1093 (21.2)
	PCI	3440 (66.8)
	CABG	614 (11.9)

Data are presented as *n* (percent). AMI—acute myocardial infarction. AV block—atrioventricular block. BMI—body mass index. CABG—coronary artery bypass surgery. CAD—coronary artery disease. CHF—congestive heart failure. COPD—chronic obstructive pulmonary disease. HbA1C—hemoglobin A1C. HDL—high-density lipoprotein. LDL—low-density lipoprotein. LM—left main coronary artery. LV—left ventricular. MI—myocardial infarction. PCI—percutaneous coronary intervention. PVD—peripheral vascular disease. STEMI—ST-elevation myocardial infarction.

**Table 2:** Cumulative incidence of new-onset type 2 diabetes mellitus by the baseline characteristics.

Parameter	Category	Cumulative Incidence	*p*-Value
**Demographics**			
Age, years	<50	0.599	0.04
	50–60	0.538	
	≥60	0.451	
Sex	Female	0.477	0.083
	Male	0.547	
Ethnicity	Jews	0.536	0.001
	Minorities (Arabs)	0.569	
**Cardiac diseases**			
Cardiomegaly	No	0.541	0.003
	Yes	0.503	
Atrial fibrillation/flutter	No	0.533	0.029
	Yes	0.63	
CHF	No	0.544	0.022
	Yes	0.55	
Chronic pulmonary heart disease	No	0.541	0.127
	Yes	0.438	
History of MI	No	0.547	0.01
	Yes	0.549	
AV block	No	0.538	0.091
	Yes	0.636	
**Cardiovascular risk factors**			
Renal diseases	No	0.542	0.322
	Yes	0.416	
Smoking	No	0.45	0.072
	Yes	0.597	
PVD	No	0.533	<0.001
	Yes	0.654	
Hypertension	No	0.52	<0.001
	Yes	0.555	
BMI, kg/m^2^	No results	0.535	<0.001
	<30	0.449	
	≥30	0.678	
**Results of laboratory tests**			
HbA1C baseline, %	No results	0.54	<0.001
	<5.7	0.416	
	5.7–6.0	0.441	
	≥6	0.701	
LDL, mg/dL	No results	0.839	<0.001
	<100	0.551	
	≥100	0.525	
Total cholesterol, mg/dL	No results	0.852	0.017
	<200	0.499	
	200–240	0.589	
	≥240	0.619	
HDL, mg/dL	No results	0.605	0.028
	<40	0.552	
	40–60	0.514	
	≥60	0.631	
Triglycerides, mg/dL	No results	0.853	<0.001
	<150	0.43	
	150–200	0.682	
	200–500	0.654	
	≥500	0.845	
**Other disorders**			
COPD	No	0.543	0.009
	Yes	0.483	
Neurological disorders	No	0.541	0.197
	Yes	0.486	
Malignancy	No	0.542	0.814
	Yes	0.336	
Anemia	No	0.556	0.341
	Yes	0.53	
	Yes	0.59	
**Clinical characteristics of AMI**			
Type of AMI	NSTEMI	0.557	<0.001
	STEMI	0.519	
**Results of echocardiography**			
Severe LV dysfunction	No	0.539	0.447
	Yes	0.432	
LV hypertrophy	No	0.535	0.022
	Yes	0.595	
Mitral regurgitation	No	0.534	0.006
	Yes	0.667	
Tricuspid regurgitation	No	0.537	0.115
	Yes	0.447	
Pulmonary hypertension	No	0.534	0.001
	Yes	0.552	
**Results of angiography**			
Measure of CAD	No or non-significant	0.498	0.462
	One vessel	0.554	
	Two vessels	0.445	
	Three vessels/LM	0.553	
	No results	0.605	0.001
**Type of treatment**			
Type of treatment	Noninvasive	0.621	0.002
	PCI	0.544	
	CABG	0.401	

AMI—acute myocardial infarction. AV block—atrioventricular block. BMI—body mass index. CABG—coronary artery bypass surgery. CAD—coronary artery disease. CHF—congestive heart failure. COPD—chronic obstructive pulmonary disease. HbA1C—hemoglobin A1C. HDL—high-density lipoprotein. LDL—low-density lipoprotein. LM—left main coronary artery. LV—left ventricular. MI—myocardial infarction. PCI—percutaneous coronary intervention. PVD—peripheral vascular disease. STEMI—ST-elevation myocardial infarction.

**Table 3:** Risk of new-onset type 2 diabetes mellitus: multivariable analysis.

Parameter	Category	B (SE)	AdjHR	(95% CI)	*p*-Value
Age, years	<50		1 (ref.)		
	50–60	0.191 (0.091)	1.21	(1.012; 1.448)	0.037
	≥60	0.031 (0.096)	1.031	(0.854; 1.244)	0.751
Ethnicity	Arabs vs. Jews	0.250 (0.083)	1.284	(1.091; 1.511)	0.003
Cardiomegaly	Yes vs. No	0.319 (0.124)	1.373	(1.078; 1.756)	0.01
History of MI	Yes vs. No	0.221 (0.098)	1.248	(1.029; 1.513)	0.024
AV block	Yes vs. No	0.479 (0.169)	1.614	(1.160; 2.246)	0.005
HbA1C baseline, %	No results	0.065 (0.139)	1.068	(0.814; 1.401)	0.637
	<5.7		1 (ref.)		
	5.7–6.0	0.654 (0.191)	1.924	(1.324; 2.795)	<0.001
	≥6.0	1.208 (0.180)	3.346	(2.353; 4.760)	<0.001
LDL, mg/dL	No results	0.526 (0.126)	1.692	(1.321; 2.167)	<0.001
	<100		1 (ref.)		
	≥100	0.235 (0.072)	1.264	(1.098; 1.455)	0.001
Hypertension	Yes vs. No	0.310 (0.070)	1.364	(1.188; 1.565)	<0.001
BMI, kg/m^2^	No results	0.120 (0.087)	1.128	(0.951; 1.337)	0.167
	<30		1 (ref.)		<0.001
	≥30	0.470 (0.128)	1.599	(1.245; 2.055)	<0.001
Smoking	Yes vs. No	0.295 (0.073)	1.343	(1.164; 1.550)	<0.001
PVD	Yes vs. No	0.337 (0.117)	1.401	(1.114; 1.761)	0.004
Type of AMI	NSTEMI vs. STEMI	0.210 (0.069)	1.233	(1.076; 1.413)	0.003
Mitral regurgitation	No results	0.329 (0.123)	1.389	(1.092; 1.768)	0.007
	No		1 (ref.)		
	Yes	0.483 (0.165)	1.622	(1.173; 2.242)	0.003

AMI—acute myocardial infarction. AdjHR—adjusted hazard ratio. AV block—atrioventricular block. B—regression coefficient. BMI—body mass index. CI—confidence interval. HbA1C—hemoglobin A1C. LDL—low-density lipoprotein. LV—left ventricular. MI—myocardial infarction. PVD—peripheral vascular disease. ref.—reference group. SE—standard error. STEMI—ST-elevation myocardial infarction.

## Data Availability

The data underlying this article will be shared upon reasonable request to the corresponding author.

## Supplementary Materials

The following supporting information can be downloaded at https://www.mdpi.com/article/10.3390/jcdd12030089/s1. Figure S1: study design. Figure S2: study flow chart. Table S1: diagnoses and interventions according to the International Classification of Diseases, Ninth Revision, Clinical Modification (ICD-9-CM) codes. Table S2: the risk score for new-onset type 2 diabetes mellitus, based on the results of a multivariable analysis. Figure S3: distribution of scores—“Pre-diabetes Sub-Scale” and “Other Risk Factors Sub-Scale”.
