# Supplementary material for: Incidence and Risk Factors for Developing Type 2 Diabetes Mellitus After Acute Myocardial Infarction—A Long-Term Follow-Up
Source: J Cardiovasc Dev Dis. 2025 Feb 28;12(3):89. doi: 10.3390/jcdd12030089 (PMC11942632; doi:10.3390/jcdd12030089)
Supplement: Supplementary file 1 [file jcdd-12-00089-s001.zip › jcdd-3383277-supplementary.pdf]

Supplemental Figure S1. Study Design.

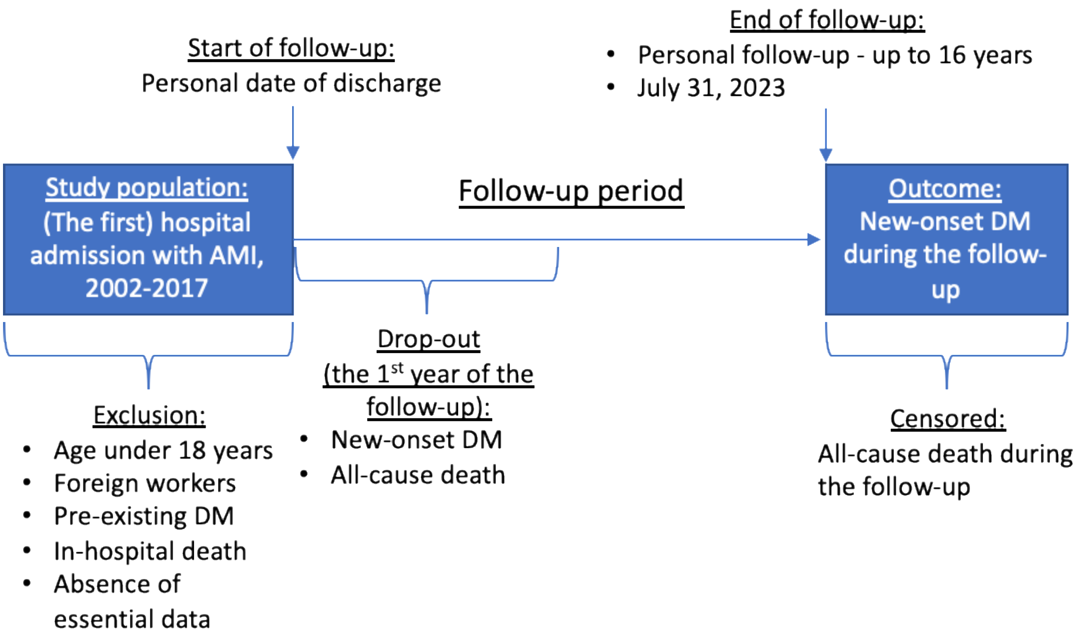

AMI – Acute myocardial infarction, DM – Diabetes mellitus

Supplemental Figure S2. Study Flow Chart.

AMI – Acute Myocardial Infarction.

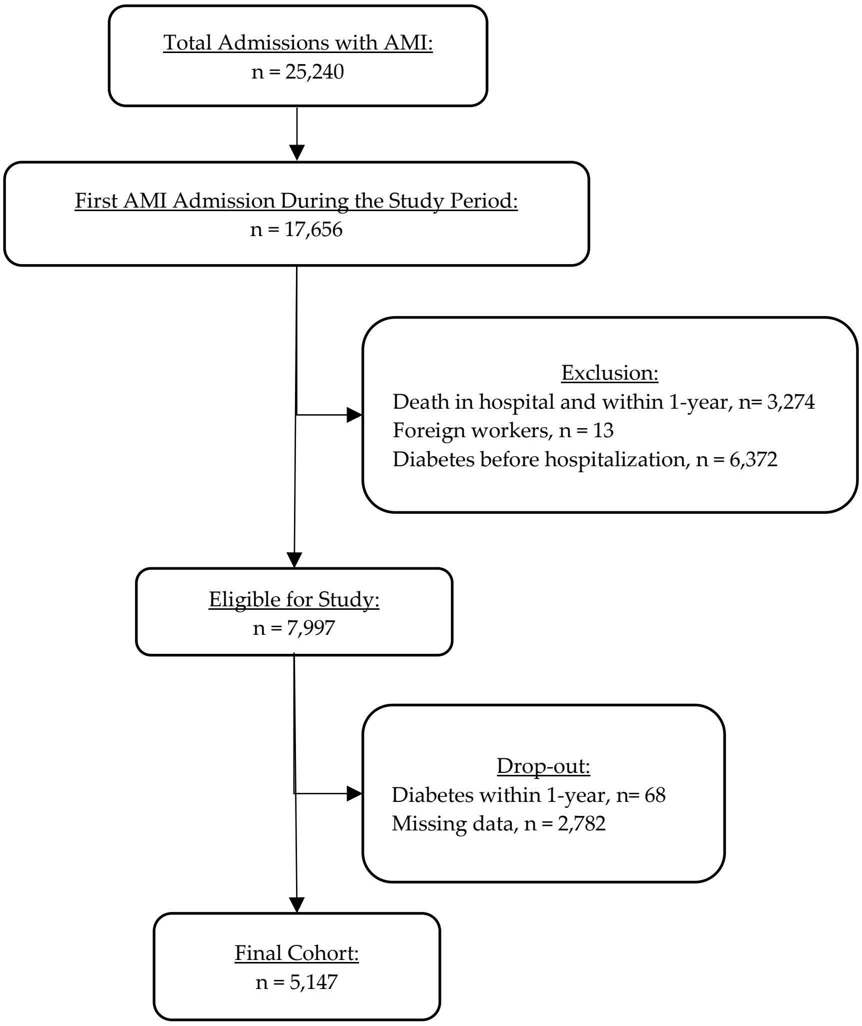

Supplemental Table S1. Diagnoses and interventions according to the International Classification of Diseases, Ninth Revision, Clinical Modification (ICD-9-CM) codes.

Diagnosis description	Diagnosis codes
Cardiomegaly	429.3
Atrial Fibrillation / Flutter	427.3
Congestive heart failure	428*
Chronic pulmonary heart disease	416*
History of myocardial infarction	412
Atrioventricular block	426.0, 426.1*
Renal diseases	585*-588*
Smoking	305.1
Peripheral vascular disease	443*, 444.2*
Hypertension	401*
Dyslipidemia	272.0-272.4
Chronic obstructive pulmonary disease	490*-496*
Neurological disorders	340-342*, 344*, 433*, 434*, 437*, 438*
Malignancy	148–208*, 230–233*
ST elevation myocardial infarction	410.0*-410.6*
Non-ST elevation myocardial infarction	410.7*-410.9*
Percutaneous coronary intervention	36.06-36.09
Coronary artery bypass surgery	36.2-26.9*

Supplemental Table S2. Risk Score for New-Onset Type 2 Diabetes Mellitus, based on the Results of Multivariable Analysis (See Table 3 in the Manuscript).

Parameter	Category	AdjHR	Weight
	**Pre-Diabetes Sub-Scale**		
HbA1C baseline, %:	No results	1.068	0
	<5.7	1 (ref.)	0
	5.7-6.0	1.924	2
	≥6.0	3.346	3
	**Other Risk Factors Sub-Scale**		
Age, years:	<50	1 (ref.)	0
	50-60	1.21	1
	≥60	1.031	0
Nationality	Arabs vs. Jews	1.284	1
Cardiomegaly	Yes vs. No	1.373	1
History of MI	Yes vs. No	1.248	1
AV block	Yes vs. No	1.614	2
LDL, mg/dl:	No results	1.692	2
	<100	1 (ref.)	0
	≥100	1.264	1
Hypertension	Yes vs. No	1.364	1
BMI, kg/m^2:	No results	1.128	0
	<30	1 (ref.)	0
	≥30	1.599	2
Smoking	Yes vs. No	1.343	1
PVD	Yes vs. No	1.401	1
Type of AMI	NSTEMI vs. STEMI	1.233	1
Mitral regurgitation:	No results	1.389	1
	No	1 (ref.)	0
	Yes	1.622	2

AMI - Acute myocardial infarction, AdjHR – Adjusted hazard ratio, AV block - Atrioventricular block, BMI - Body mass index, HbA1C - Hemoglobin A1C, LDL - Low density lipoprotein, MI - Myocardial infarction, NSTEMI – non-ST elevation myocardial infarction; PVD - Peripheral vascular disease, ref. – Reference group, STEMI - ST elevation myocardial infarction.

Supplemental Figure S3. Distribution of Scores - 'Pre-Diabetes Sub-Scale' and 'Other Risk Factors Sub-Scale'.

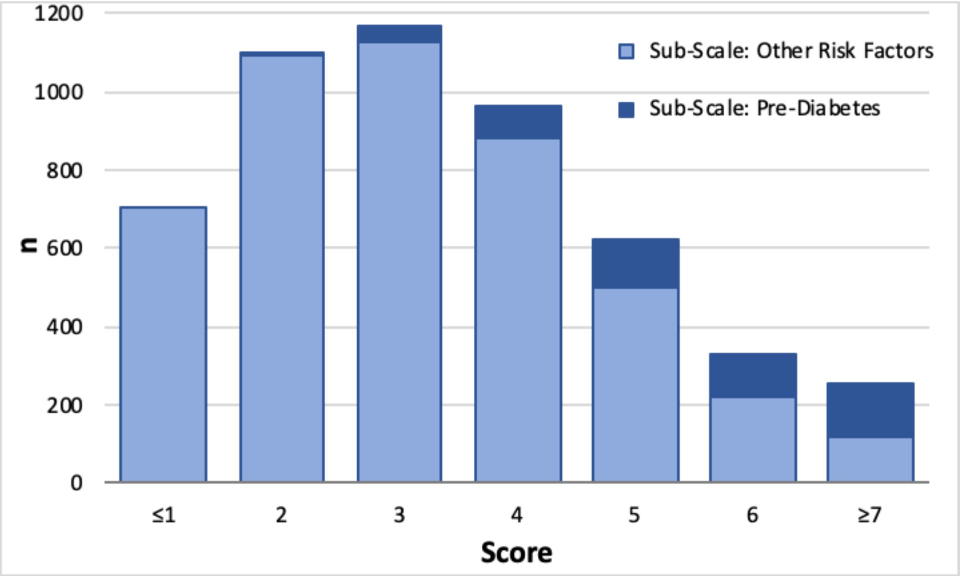

'Pre-diabetes sub-scale' refers to hemoglobin A1C levels ≥5.7%. 'Other risk factors sub-scale' consisted of age, ethnicity, low-density lipoprotein, hypertension, body mass index, smoking, cardiomegaly, history of myocardial infarction, type of acute myocardial infarction, atrioventricular block, peripheral vascular disease and mitral regurgitation (see Supplemental Table S2).
